# The Effect of a Supplier’s Eco-Design on the Economic Benefits of a Supply Chain and Associated Coordination

**DOI:** 10.3390/ijerph182413357

**Published:** 2021-12-18

**Authors:** Junjun Liu, Yong Geng, Biao Chen, Xiqiang Xia

**Affiliations:** 1 Faculty of Business Administration, School of Business Administration, Southwestern University of Finance and Economics, Chengdu 611130, China; liujunj@swufe.edu.cn; 2 College of Environmental Science and Engineering, Shanghai Jiao Tong University, Shanghai 200240, China; ygeng@sjtu.edu.cn; 3 Business School, Zhengzhou University, Zhengzhou 450001, China; xqxia@zzu.edu.cn

**Keywords:** eco-design, green supply chain, supply chain coordination, game theory

## Abstract

The eco-design of upstream suppliers can reduce the environmental impact from the production process for downstream customers. To analyze the effect of suppliers’ eco-design on the economic benefits of up-downstream supply chain and the mechanisms, this study constructed a master–slave game theory model for a supplier and a manufacturer. Based on this game theory model, this study comparatively analyzes the effects on raw material/part prices, retail product prices, sale volume, revenue, and eco-design effort level under three conditions (no eco-design, decentralized decision-making with eco-design, centralized decision-making with eco-design). And to further analyze the effect of eco-design costs on the optimal solution, this article takes the supply chain of tire production as an example. This analysis could provide suggestions for the suppliers and manufacturers to develop and improve their eco-design. The main results are as follows: the supplier eco-design is beneficial to improving the overall economic benefits for suppliers and manufacturers under certain conditions, and the range in which a supplier is willing to implement eco-design in a decentralized decision-making situation is wider than that in a centralized decision-making situation; when a supplier implements an eco-design, it will transfer part of the cost to the manufacturer by raising the unit raw material/parts prices. Meanwhile, the manufacturer can reduce the production cost when the benefit of eco-design is more than the increased purchasing price, and they can decrease the retail price to expand the sales volume. Hence, consumers will benefit from lower prices. Thus, it is a multi-win situation among the suppliers, manufacturers, and consumers.

## 1. Introduction

More than 80% of the environmental impact of the whole product life-cycle is determined by the design stage [1]. Therefore, more and more companies have begun to pay attention to eco-design as the increasing pressure of environmental and ecological protection [2,3]. Eco-design can effectively reduce material consumption, energy consumption, and pollutant emission during the processes such as product production, use, transportation, recycling, and reuse. For parts of high energy consumption and heavy pollution manufacturing companies, the production process is the stage of direct resource and energy consumption and the main stage of the whole product life-cycle to produce pollutant emissions [4]. For example, according to the data of the Chinese National Bureau of Statistics, the electricity consumption of high-energy-consuming companies accounted for about 30% of the total electricity consumption in 2018 nationwide [5]. According to the data from the U.S. Bureau of Energy Information and Statistics, the energy consumption in the production process of manufacturing companies accounts for about 80% of their total energy consumption on average, of which the operation of production equipment accounts for 48.2%. The function of other auxiliary equipment such as heating and cooling accounts for more than 30% [6]. Therefore, the ‘Guiding Opinions on the Development of Eco-Design of Industrial Products’, a document issued by the Chinese government, is to encourage companies to carry out eco-design. It clearly states that when carrying out eco-design, manufacturers need to focus on reducing resource and energy consumption and the discharge of pollutants and toxic and hazardous substances in the production process (http://www.gov.cn/zwgk/2013-02/27/content_2341028.htm, accessed on 27 February 2013). However, if companies only rely on their own eco-design efforts to continuously promote energy conservation and emissions reduction, the effects and implementation space are limited. Hence, they need to cooperate with the up-down stream of the supply chain. And it can generate potential opportunities for energy conservation and emissions reduction, and achieve good results [7,8].

Eco-design is significant for upstream and downstream companies in the supply chain to achieve environmental and economic externalities [9,10], and is one of the important practices of green supply-chain management. Suppliers’ eco-design efforts for raw materials and/or parts (referred to as ‘raw materials’) will impact the production, use, and end-of-life treatment of downstream customers, namely manufacturers [11]. The eco-design efforts of suppliers to the production process of downstream products can significantly reduce raw material consumption, energy consumption, and pollutant emission [12,13]. For example, the optimized design and material selection of parts suppliers in the electromechanical industry will significantly reduce the energy consumption and waste in the product production process (e.g., upstream eco-design can reduce the further processing of some parts, such as cutting and welding) [14,15]. In the rubber industry, the development of new additives and auxiliaries can significantly reduce the material and energy consumption in the tire production process, and reduce the production cost per unit product [16]. However, the eco-design of suppliers will increase their cost besides benefit downstream manufacturers. And the supplier’s eco-design efforts may face the risk of no one paying. For manufacturers, they need to weigh the cost of input and the profit of cooperation. It is the crucial factor to decide whether to incentivize suppliers through cost-sharing and revenue-sharing [17]. Therefore, based on the game theory perspective, this study explores whether the upstream and downstream companies of the green supply chain should cooperate in the eco-design of the production process, and how to realize green supply chain performance coordination during the cooperation process. It is of great practical significance.

The existing literature on the eco-design of supply chain mainly focused on the improving of the greenness of downstream products through the suppliers’ eco-design of raw materials and parts [18], or the improving the recyclability at the end-of-life of products [19]. To explore the role of suppliers’ eco-design on the economic benefits (e.g., energy conservation and emission reduction) for the production process of manufacturers (referred to as ‘eco-design’), this study constructs three game theory situations composed of one supplier and one manufacturer (no eco-design, eco-design in decentralized decision-making and eco-design in centralized decision-making). And then it compares and analyzes the changes in eco-design efforts, unit retail product price, demand and profit in each case. Finally, it proposes an upstream and downstream (supplier-manufacturer) eco-design coordination mechanism (revenue-sharing and cost-sharing). The goal is to provide a scientific decision-making basis for upstream and downstream companies to choose the optimal eco-design mode and cooperation mechanism.

## 2. Literature Review

**Figure 1:**
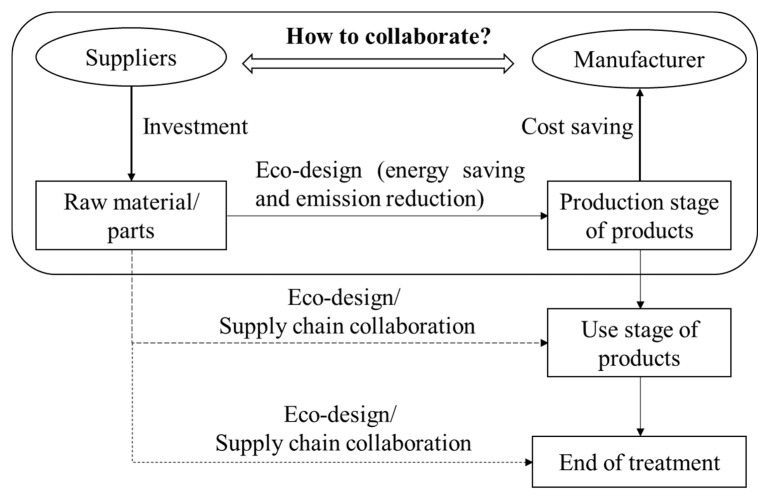
Eco-design models for the reduction of energy consumption and pollutant emission in the downstream production process.

The literature related to this study mainly involves two aspects: one is the research on eco-design in the supply chain, and the other is the research on the coordination of the green supply chain. The research on eco-design in the supply chain mainly focused on the eco-design collaboration for the product-use and end-treatment stages (e.g., remanufacturing). The research on the green supply chain coordination is primarily aimed at the benefit-sharing and cost-sharing issues after the improvement of a product’s greenness. And as the emergence of an extended producer responsibility system, it explored the supply chain coordination for the end-treatment of products. Based on this research status, this study proposes the eco-design model that explains how suppliers save energy and reduce emissions in the downstream production process, as shown in Figure 1 (the solid line diagrams represent this study’s content).

The eco-design behavior of manufacturers is mainly based on the pressure of external stakeholders and the needs of internal development, and its goal is to achieve environmental performance and obtain more economic benefits by forming market competitiveness [20,21]. And the eco-design is useful to each stage of the product life-cycle (product manufacturing, use and end-treatment). As we all know, the different stages match with different supply chain members, and the cooperation among them is crucial to achieving the goal of eco-design [22,23]. Zhu and He [18] consider that the supply chain competition may have effects on the greenness in product design. Furthermore, around the issues of greenness, Mohammed et al. [24] explored the greenness of manufacturers’ product designing under carbon taxes and government subsidies, and Li et al. [7] discussed how manufacturers encourage suppliers to cooperate in eco-design to improve the greenness of a product and increase revenue. For the needs of internal development, Subramanian et al. [19] pointed out that the cooperation in product eco-design between manufacturers and retailers under the extended producer responsibility system is beneficial to reduce the environmental impact of the use process and improve re-manufacturability. Based on their research, Cao et al. [25] discussed the effect of an extended producer responsibility system on the cooperation in product eco-design between manufacturers and downstream customers, and their study has taken the environmental protection and recyclability of products into consideration to achieve a deeper analysis goal. Others such as Gu et al. [26] discussed the importance of the cooperation between manufacturers and remanufacturers for the higher profits, and Wang et al. [27] revealed the impact of different ways of bearing remanufacturing design costs on environmental performance and benefits.

Furthermore, the researches on the cooperation between upstream and downstream in eco-design involve the coordination of the supply chain, and mainly focus on green (environmental protection) and low-carbon. The core content discussed how to improve the greenness of products through bilateral efforts to increase sales and benefit both partners. The first is the research on the supply chain coordination related to green (environmental protection). As Cosimato and Troisi [28] pointed out that green related technologies play an important role for firms to gain competitive advantage. Hence, Yang et al. [29] considered the effects of joint green R&D between supplier-manufacturers on market demand from the perspective of time dynamics. Based on the results, they compared and analyzed R&D strategies under wholesale price contracts, revenue sharing contracts and centralized decision-making conditions. Based on the consumer side to explore green R&D, Song and Gao [30] discussed the supply chain game theory model of product greenness under different decision-making situations, and they found the impact of revenue-sharing contracts on product greenness and profit. Focused on high energy-consuming companies, Ouyang and Ju [31] analyzed the forming conditions of self-energy saving and revenue-sharing energy-saving modes in coordinated and uncoordinated contexts. For example, in an uncoordinated context, manufacturers tend to choose the individual energy-saving mode if the shared energy-saving benefits are lower than the individual energy-saving benefits. Furthermore, Chan et al. [32] discussed the synchronous coordination model of suppliers and manufacturers in reverse logistics, and found that this model has better economic and environmental performance than the independent model. Wu and Kao [33] discussed the choice of original manufacturer on its outsource remanufacturing business, and explained its effect on supply-chain benefits and the coordination mechanisms. The second is the research on low-carbon related supply chain coordination. Liu and Song [34] discussed the effects of the adjustment of the government’s carbon tax rate on the cooperative R&D in the supply chain under the background of low carbon, and found that it can realize the coordination of the supply chain for energy conservation and emissions reduction. Similarly, Park et al. [35] discussed the impact of the carbon tax on the coordination of interests between retailers and consumers based on the maximization of social welfare. In order to analyze the relationship between input cost and emission reduction efficiency under different low-carbon technology characteristics in detail, Yang et al. [36] established a dynamic optimization model of supply chain cooperation and found that low-carbon technology characteristics under certain conditions can achieve a win-win situation of supply chain environment and economic performance. Xia et al. [37] explored the forming conditions and functions of carbon emissions reduction cost sharing contracts to coordinate the profit distribution of supply chain. Bai et al. [38] analyzed the balance of investment strategies for emission reduction of supply chain companies in the low-carbon market, and found that different intensities of willingness to share cost produce different behavior choices. Yi and Li [39] analyzed the impact of carbon-tax and energy-saving subsidy policies on manufacturers’ decision-making, and pointed out that a cost-sharing contract can promote the formation of upstream and downstream cooperation mechanisms in the supply chain, and maximize the effect of energy conservation and emissions reduction.

Based on the literature review above, the research on supply-chain eco-design and its coordination mechanisms have formed relevant results [7,40]. Still, the research related to eco-design under the background of the supply chain is focused on the stage of product use and product recycling at end-of-lifecycle. Few studies focused on the product production process. Suppliers’ eco-design efforts for product production process will impact the production, use, and end-of-life treatment of manufacturers. However, the eco-design of suppliers will increase their cost and may face the risk of no one paying. Therefore, this study explored the following research questions to solve the above problem. First, what are the boundary conditions for suppliers to implement eco-design to benefit both upstream and downstream? Second, what are the supplier’s eco-design efforts and benefits of supply chain under the different game conditions? Third, to achieve the overall optimization of upstream and downstream, how the upstream and downstream can stimulate suppliers’ efforts through the coordination mechanism.

To solve the problems above, this paper constructs an upstream and downstream green supply chain game model based on upstream eco-design. By comparing and analyzing the benefits when there is no eco-design and decentralized decision-making with eco-design, we suggest that eco-design is conducive to improving the boundary of upstream and downstream benefits. Furthermore, we compare and analyze the size of the optimal solution of each variable in the three game situations (no eco-design, decentralized decision-making with eco-design, centralized decision-making with eco-design), and propose the effects of different situations on the optimal solution. Finally, we suggest that a revenue-sharing and cost-sharing coordination mechanism can encourage supplier eco-design behavior, and achieve the overall optimization of upstream and downstream.

## 3. Model Formulation

### 3.1. Problem Description

**Figure 2:**
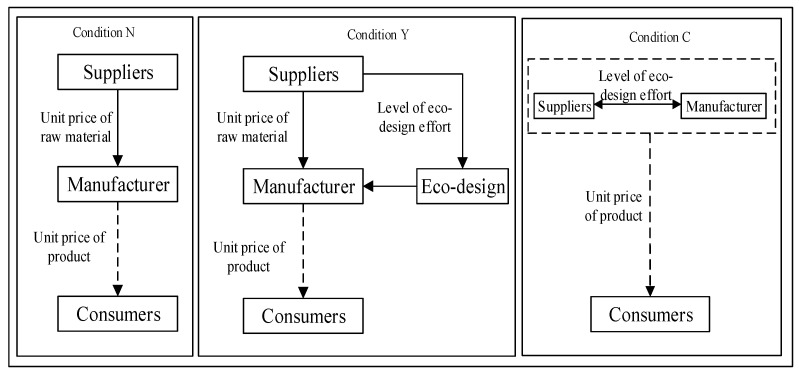
Schematic diagram of the decision-making game theory model between suppliers and manufacturers.

To improve the economic benefits of the supply chain, this article explores whether a supplier should implement eco-design for the production process for a customer (manufacturer) product. We discuss changes in economic benefits in three conditions based on a supply chain consisting of a supplier and a manufacturer as Figure 2. 

To analyze the impact of eco-design on suppliers and manufacturers, firstly, we establish a game model between suppliers and manufacturers in no eco-design, that is no eco-design condition. Secondly, we build a game model between suppliers and manufacturers with eco-design, that is eco-design condition. Finally, to design an eco-design contract for the supplier and manufacturer when they cooperate, it is necessary to study the centralized decision-making condition, that is, the integration of the supplier and the manufacturer. Suppliers implement eco-design around the raw materials they supply to reduce material consumption, energy consumption, and pollutant emissions in the production process of downstream products. The supplier eco-design has a buoyant external economy for the downstream manufacturer. However, it means cost input for suppliers. The critical premise for achieving supply chain coordination and optimal performance is that both parties can improve economic benefits. Therefore, manufacturers need to encourage suppliers to implement eco-design, through strategies such as cost-sharing. 

Under no eco-design condition, the focus of the game between the supplier and the manufacturer is unit purchase price of raw material. In other words, the game focus between the supplier and the manufacturer is the unit price of raw material. The manufacturer decides the unit retail price of products based on the unit purchase price of raw material. The supplier will play a game with the manufacturer on the unit price of raw material based on the raw material production cost and the eco-design cost in the stage of design for production. And the manufacturer decides the unit retail price of products based on the unit price of raw material and the cost saving of eco-design. In centralized decision-making condition, the manufacturer and the supplier are merged into one. Hence, the manufacturer decides the unit retail price of products based on production cost, eco-design cost and cost saving.

When the eco-design is not implemented in the initial condition, the supplier first determines the unit retail price of raw materials. And then the manufacturer determines the unit retail price of products based on the unit purchase price of raw material. When eco-design is implemented, the supplier first determines the effort level of eco-design and the unit retail price of products. Then, the manufacturer determines the unit retail price of products based on the unit purchase price of raw material and the effort level of eco-design of unit raw material. However, it should be noted that when the supplier acts as an initiator, the manufacturer as a beneficiary may choose a negative strategy such as simply buying raw material without sharing the cost. Therefore, to promote final collaboration (suppliers implementing eco-design for raw materials and manufacturers sharing cost), it needs to design a revenue-sharing and cost-sharing contract to coordinate. The content of eco-design, decision sequence and its variables are described in Table 1.

### 3.2. Notation Description

N: The condition of no eco-design;

Y: The condition of decentralized decision-making with eco-design;

C: The condition of centralized decision-making with eco-design;

$c_{s}$: The supplier’s production cost per unit raw material; 

$c_{m}$: The manufacturer’ production cost per unit product;

δ: The profit coefficient per unit product obtained by the manufacturer due to the supplier eco-design effort level. This refers to the proportion of cost reduced by the reduction of raw material consumption, energy consumption and pollutant emission in the product production process; 

Q: The demand quantity of products in the market;

α: The sensitivity of consumers to the unit retail price of products;

$p_{i}$: The unit retail price of products in condition, where; 

$q_{i}$: The sales volume of products in condition, where;

$\tau_{i}$: The effort level of eco-design in condition, where;

$w_{i}$: The unit purchase price of raw material in condition, where;

$\pi_{iS}$: The economic benefits of the supplier in condition, where;

$\pi_{iM}$, The economic benefits of manufacturers in condition, where;

$\pi_{C}$: Total revenue of supply chain under centralized decision-making situation.

### 3.3. Hypotheses

To ensure the scientificity of the model, the market demand function adopted in this study is a classic function, see Hypothesis 1. On the other hand, although for the manufacturer, there may be many suppliers. This study takes the suppliers as a whole to supply parts based on the existing literature, see Hypothesis 2. Finally, a classic cost function was adopted to describe the relationship between the cost of eco-design and the degree of eco-design efforts, that is, the cost of eco-design is a quadratic function of the degree of eco-design efforts, see Hypothesis 3.

Based on relevant literature and the actual situation, this article puts forward the following hypotheses:

**Hypothesis** **1.** *As the supplier implements raw material eco-design for the product production process for customers, it is beneficial for the reduction of energy consumption and pollutant emission for the downstream production process. However, it has no negative effects on the stability of product quality, production capacity and production flow. Meanwhile, as it focuses on the production process for products, it has no effect on product greenness. Hence, the demand volume of products for the manufacturer is only affected by price* [41] *namely* q=Q−αp.

**Hypothesis** **2.** *Referring to the study of Liu and Song* [34], *the relationship between the raw materials provided by the supplier and the final product quantity produced by the manufacturer is assumed as 1:1; that is, when the manufacturer produces a unit of product, it needs to consume a unit of raw material provided by the supplier. In practice, if it is not 1:1, it still has no effect on the results of this study. The model established in this study can be extended to a game theory model between multiple suppliers and one manufacturer. For convenience of the research, this study assumes that the relationship between the raw materials provided by the supplier and the final product quantity produced by the manufacturer is 1:1.*

**Hypothesis** **3.** *There is a positive correlation between the cost of implementing eco-design and the effort level. Based on the research of Xia et al.* [37], *the cost of the supplier eco-design is $\frac{k\tau_{i}^{2}}{2}$, where i∈{Y,C} and k represents the cost coefficient of the supplier’ s eco-design.*

The demand function is the same as literature [41]. Meanwhile, this research only studies one supplier and one manufacturer, which is the same as literature [34]. However, the impact of carbon emission reduction efforts on the demand for low-carbon products is q=Q−αp+βτ in literature [37]. where, τ, β respectively represent the carbon emission reduction efforts and the sensitivity coefficients of consumers’ carbon emission reduction efforts. In this article, the impact of eco-design on manufacturers is represented by the saving of production costs.

## 4. Model Formulation and Solutions

### 4.1. Model Formulation 

In the case that the supplier implements no eco-design:

(1) $$\pi_{NS}=(w_{N}-c_{s})(Q-\alpha p_{N})$$

(2) $$\pi_{NM}=(p_{N}-w_{N}-c_{m})(Q-\alpha p_{N})$$

In the case that the supplier implements eco-design:

(3) $$\pi_{YS}=(w_{Y}-c_{s})(Q-\alpha p_{Y})-\frac{k\tau_{Y}^{2}}{2}$$

(4) $$\pi_{YM}=(p_{Y}-w_{Y}-c_{m}+\delta \tau_{Y})(Q-\alpha p_{Y})$$

In the case of centralized decision-making:

(5) $$\pi_{C}=(p_{C}-c_{m}-c_{s}+\delta \tau_{C})(Q-\alpha p_{C})-\frac{k\tau_{C}^{2}}{2}$$

### 4.2. Model Solutions

To obtain the optimal solution, Lemma 1 is as follows.

**Lemma** **1.** (i) Equation (2) with respect to $p_{N}$ is a concave function. The optimal solution $p_{N}^{*}$ is obtained by Equation (2), and it is substituted into Equation (1). Equation (1) with respect to $w_{N}$ is a concave function.

(ii) Equation (4) with respect to $p_{Y}$ is a concave function. The optimal solution $p_{Y}^{*}$ is obtained by Equation (2), and it is substituted into Equation (3). Equation (3) with respect to $w_{Y},\;\tau_{Y}$ is a concave function.

(iii) Equation (5) with respect to $p_{C},\;\tau_{C}$ is a concave function.

**Proof.**  The proofs of (i) and (iii) are simpler than (ii). Hence, only the proof of (ii) is given here. The specific contents are as follows:

Regarding Equation (4) with respect to $p_{Y}$, we calculate the first-order and second-order derivatives:

(6) $$\frac{\partial \pi_{YM}}{\partial p_{Y}}=Q-2\alpha p_{Y}+\alpha w_{Y}+\alpha c_{m}-\alpha \delta \tau_{Y}$$

(7) $$\frac{\partial^{2}\pi_{YM}}{\partial p_{Y}{}^{2}}=-2\alpha$$

We can see from Equation (7): $\frac{\partial^{2}\pi_{YM}}{\partial p_{Y}{}^{2}}=-2\alpha <0$, that Equation (4) $p_{Y}$ is a concave function. Let Equation (6) be equal to zero: $p_{Y}^{*}=\frac{Q+\alpha w_{Y}+\alpha c_{m}-\alpha \delta \tau_{Y}}{2\alpha }$, and substitute $p_{Y}^{*}$ into Equation (3):

(8) $$\pi_{YS}=\frac{(w_{Y}-c_{s})(Q-\alpha w_{Y}-\alpha c_{m}+\alpha \delta \tau_{Y})-k\tau_{Y}^{2}}{2}$$

Regarding Equation (8) with respect to $w_{Y},\;\tau_{Y}$, we calculate the first-order and second-order derivatives:

(9) $$\frac{{\partial \pi }_{YS}}{\partial w_{Y}}=\frac{Q-2\alpha w_{Y}-\alpha c_{m}+\alpha c_{s}+\alpha \delta \tau_{Y}}{2}$$

(10) $$\frac{\partial \pi_{YS}}{\partial \tau_{Y}}=\frac{\alpha \delta (w_{Y}-c_{s})-2k\tau_{Y}}{2}$$

(11) $$\frac{\partial^{2}\pi_{YS}}{\partial \tau_{Y}\partial w_{Y}}=\frac{\partial^{2}\pi_{YS}}{\partial w_{Y}\partial \tau_{Y}}=\frac{\alpha \delta }{2}$$

(12) $$\frac{\partial^{2}\pi_{YS}}{\partial w_{Y}{}^{2}}=-\alpha ,\;\frac{\partial^{2}\pi_{YS}}{\partial \tau_{Y}{}^{2}}=-k$$

We can see from Equation (9) to Equation (12) that the Hessian matrix of $w_{Y},\;\tau_{Y}$ is as follows:

(13) $$H=\left[\begin{array}{cc} -\alpha & \frac{\alpha \delta }{2}\\ \frac{\alpha \delta }{2} & -k \end{array}\right]$$

Based on Equation (13), we can get Equation (14):

(14) $$-\alpha <0,\;\left|H\right|=\frac{\alpha (4k-\alpha \delta^{2})}{4}>0$$

Hence, Equation (8) with respect to $w_{Y},\;\tau_{Y}$ is a concave function. Thus, (ii) is proved.

The proof of Lemma 1 is completed. □

Based on Lemma 1, we can see the values of the variables in three eco-design situations. The detailed content is described in Conclusion 1.

**Conclusion** **1.** The comparison of the optimal solutions of variables in three eco-design situations is as follows. See Table 2.

## 5. Model Analysis

Based on Conclusion 1, the boundary conditions for supplier’s eco-design are obtained. The detailed content is described in Conclusion 2.

**Conclusion** **2.** In the case of decentralized and centralized decision-making, the condition for implementing eco-design is that the benefits of suppliers and manufacturers are positive. If the eco-design effort level is greater than zero, the supplier has implemented eco-design activities. Hence, the boundary conditions of supplier eco-design are as follows:

(i) In the condition of decentralized decision-making: $\frac{\delta (Q-\alpha c_{m}-\alpha c_{s})}{4k-\alpha \delta^{2}}>0\Leftrightarrow 4k-\alpha \delta^{2}>0\Leftrightarrow \frac{k}{\delta^{2}}>\frac{\alpha }{4}$. Hence, if $\frac{k}{\delta^{2}}>\frac{\alpha }{4}$, suppliers are willing to implement eco-design. Otherwise, they are unwilling to implement eco-design
(ii) In the condition of centralized decision-making: $\frac{\delta (Q-\alpha c_{m}-\alpha c_{s})}{2k-\alpha \delta^{2}}>0\Leftrightarrow 2k-\alpha \delta^{2}\Leftrightarrow \frac{k}{\delta^{2}}>\frac{\alpha }{2}$. Hence, if $\frac{k}{\delta^{2}}>\frac{\alpha }{2}$, suppliers are willing to implement eco-design. Otherwise, they are unwilling to implement eco-design.

Conclusion 2 shows that when consumers’ sensitivity to the unit retail price per product is constant, the ratio of the eco-design cost coefficient and the square of the cost savings per unit product as the eco-design for the manufacturer is higher than a threshold value (the threshold value is the multiple of consumers’ sensitivity to the unit retail price of products). If the supplier eco-design effort level is greater than zero, the supplier chooses to implement eco-design. Otherwise, the supplier does not implement eco-design. Based on the boundary conditions above, compared to centralized decision-making, suppliers are willing to implement eco-design in a larger area under a decentralized decision-making condition.

The threshold value is the multiple of consumers’ sensitivity to the unit retail price of products; that is, when the sensitivity of consumers to the unit retail price of a product is higher, the threshold value is higher. Hence, it is suggested to reduce the sensitivity of consumers to the unit retail price of a product, such as by advertisements, promoting other functions of products and building an environment-friendly image, etc.

Management suggestion 1: The threshold for eco-design is related to consumers’ sensitivity to the unit price of products. Therefore, manufacturers or parts suppliers should reduce consumers’ sensitivity to the unit price of products. And it can indirectly reduce the threshold. For example, as advertising can increase consumer’ awareness of environmental protection, it can indirectly reduce consumers’ sensitivity to the unit price of products.

**Conclusion** **3.** The effects of eco-design on unit raw material price, unit retail product price and demand volume are:

(i) $w_{N}^{*}<w_{Y}^{*}$;
(ii) $p_{C}^{*}<p_{Y}^{*}<p_{N}^{*}$;
(iii) $q_{N}^{*}<q_{Y}^{*}<q_{C}^{*}$

**Proof.**  (i) $w_{Y}^{*}-w_{N}^{*}=\frac{\delta^{2}(Q-\alpha c_{m}-\alpha c_{s})}{2(4k-\alpha \delta^{2})}>0$, that is, $w_{N}^{*}<w_{Y}^{*}$.

(ii) $p_{N}^{*}-p_{Y}^{*}=\frac{\delta^{2}(Q-\alpha c_{m}-\alpha c_{s})}{4(4k-\alpha \delta^{2})}>0$ that is, $p_{Y}^{*}<p_{N}^{*}$;

$p_{Y}^{*}-p_{C}^{*}=\frac{2k^{2}(Q-\alpha c_{m}-\alpha c_{s})}{\alpha (2k-\alpha \delta^{2})(4k-\alpha \delta^{2})}>0$, that is, $p_{C}^{*}<p_{Y}^{*}$, hence, $p_{C}^{*}<p_{Y}^{*}<p_{N}^{*}$.

Similarly, (iii) could be proved. □

**Conclusion** **4.** Changes in the degree of eco-design effort level in different conditions: $\tau_{Y}^{*}<\tau_{C}^{*}$.

**Proof.**  $\tau_{C}^{*}-\tau_{Y}^{*}=\frac{2\delta \kappa (Q-\alpha c_{m}-\alpha c_{s})}{\left(2k-\alpha \delta^{2})(4k-\alpha \delta^{2}\right)}>0$, hence, $\tau_{Y}^{*}<\tau_{C}^{*}$. □

Conclusion 4 shows that the optimal degree of eco-design effort in centralized decision-making is higher than in decentralized decision-making. Taking Conclusion 3 into consideration, it can be seen that when suppliers implement eco-design, they will transfer part of the eco-design cost to manufacturers by increasing the price of raw materials per unit. Theoretically, as suppliers implement eco-design and increase the purchase price of raw materials per unit, compared to no eco-design, manufacturers should increase their unit product sales prices to compensate for the increased cost of raw material purchase prices. However, in practice, the unit revenue brought by suppliers to implement eco-design can make up or even exceed the increased unit raw material cost of manufacturers, which implies that the production cost of manufacturers is lower than the situation of no eco-design. Thus, the manufacturer has a certain room for price reduction. Taking the sensitivity of sales volume to prices into account, manufacturers will promote the sales volume of their products by reducing prices, and consumers will benefit from the price reduction.

In a centralized decision-making condition, as the product sales model is similar to ‘direct selling’, it can avoid the ‘marginal effect’ of the supply chain and make unit product sales price the smallest. However, the greatest eco-design effort level leads to the largest product sales volume in a centralized decision-making situation.

Management suggestion 2: The degree of eco-design efforts is related to the decision-making mode. That is, the eco-design in centralized decision-making is greater than that in decentralized decision-making. Therefore, suppliers and manufacturers should cooperate to avoid the "marginal effects" caused by non-cooperation, such as the revenue-sharing and cost-sharing contract proposed in this study.

**Conclusion** **5.** The effects of eco-design on revenue: $\pi_{NS}^{*}<\pi_{YS}^{*},\;\pi_{NM}^{*}<\pi_{YM}^{*}$

**Proof.**  $\pi_{YS}^{*}-\pi_{NS}^{*}=\frac{\delta^{2}{\left(Q-\alpha c_{m}-\alpha c_{s}\right)}^{2}}{8(4k-\alpha \delta^{2})}>0$, that is, $\pi_{NS}^{*}<\pi_{YS}^{*}$;

$\pi_{YM}^{*}-\pi_{NM}^{*}=\frac{\alpha \delta^{2}{\left(Q-\alpha c_{m}-\alpha c_{s}\right)}^{2}(8k-\alpha \delta^{2})}{16\alpha {\left(4k-\alpha \delta^{2}\right)}^{2}}>0$, that is, $\pi_{NM}^{*}<\pi_{YM}^{*}$. □

Hence, Conclusion 5 is proved.

Combined with Conclusions 3 and 4, Conclusion 5 shows that the revenue of both the supplier and the manufacturer can be improved in supplier’s eco-design condition. If the supplier implements eco-design, the revenue of both the supplier and the manufacturer is higher than that in the condition of no eco-design. Conclusion 5 shows the feasibility of this eco-design model.

The above conclusions indicate: if it satisfies boundary of these conclusions, suppliers in a decentralized decision-making condition can choose to increase the price of raw materials to compensate for the cost of eco-design input, and they can improve their revenue. However, in practice, although suppliers have the motivation to implement eco-design, their enthusiasm for eco-design will be affected by the existence of eco-design risks, funding gaps, and the manufacturer’s acceptance after raising the price of raw materials. For the manufacturer, if the supplier implements the eco-design, the optimal retail price will be lower. It can not only promote the increase in sales volume and revenue but also bring value to consumers. Based on the above analysis, it shows that the supplier eco-design unexpectedly forms a win-win situation, in which suppliers, manufacturers, and consumers benefit from each other. When the manufacturer realizes the environmental and economic benefits of energy conservation brought about by the supplier eco-design, it is necessary to design reasonable contracts (such as revenue-sharing and cost-sharing) to encourage supplier eco-design efforts. Hence, the eco-design model can be formed and operated.

In order to facilitate the discussion of the supply chain coordination mechanism under centralized decision-making and decentralized decision-making conditions:

$$\pi_{Y}^{*}=\pi_{YS}^{*}+\pi_{YM}^{*}=\frac{k{\left(Q-\alpha c_{m}-\alpha c_{s}\right)}^{2}(6k-\alpha \delta^{2})}{2\alpha {\left(4k-\alpha \delta^{2}\right)}^{2}}\;$$

**Conclusion** **6.** The total revenue of centralized decision-making is greater than that of decentralized decision-making in the eco-design condition, that is $\pi_{Y}^{*}<\pi_{C}^{*}$.

**Proof.**  $\pi_{C}^{*}-\pi_{Y}^{*}=\frac{2k^{3}{\left(Q-\alpha c_{m}-\alpha c_{s}\right)}^{2}}{\alpha (2k-\alpha \delta^{2}){\left(4k-\alpha \delta^{2}\right)}^{2}}>0$, that is, $\pi_{Y}^{*}<\pi_{C}^{*}$. □

Conclusion 6 shows that the total revenue of centralized decision-making is greater than that of decentralized decision-making in the eco-design condition; that is, centralized decision-making can avoid the ‘marginal effect’ and reduce the loss of upstream and downstream revenue. The result is that it can increase the revenue of centralized decision-making. Hence, this conclusion further proves the necessity of supply chain coordination to achieve overall optimality.

Management suggestion 3: Although the eco-design of suppliers can increase their component costs, manufacturers will increase the unit price of the supplying price of parts to make up for the eco-design cost. And it ultimately increases the supplier’s revenue. The revenue of suppliers and manufacturers is also affected by the decision-making mode. Therefore, manufacturers and suppliers should strengthen cooperation and increase the degree of eco-design efforts.

In order to allow the revenue of suppliers and manufacturers to achieve overall optimality, this research proposes a coordination mechanism based on the revenue-sharing contract. In decentralized decision-making with the eco-design condition, the profits of suppliers and manufacturers are retained. As the profits of centralized decision-making are higher than those of decentralized decision-making, this research allocates a higher portion than the retained profit through a revenue-sharing contract. The result is that the overall supply chain benefit achieves optimality. In a centralized decision-making condition, the proportion of the overall supply chain profit of the supplier is β, and the proportion of the manufacturer is 1−β. According to the contract, the revenue-sharing contract implies that when the supplier supplies raw materials, the unit price of the raw material is $w_{C}=(1-\beta )(p_{C}-c_{m}+\delta \tau_{c})+\beta c_{s}$. Hence, according to the contract, the maximum price of raw materials to the supplier is $p_{C}-c_{m}+\delta \tau_{c}$, and the minimum price is $c_{s}$. Generally, the pricing is in a minimum and maximum price range. The mathematical formula is $(1-\beta )(p_{C}-c_{m}+\delta \tau_{c})+\beta c_{s}$, and it is referred to as $w_{C}$.

According to the contract, the supplier’s price of raw materials is lower than the price $w_{C}$ in a decentralized decision-making condition. The manufacturer needs to distribute the increased revenue to the supplier according to the stipulated proportion of the contract, and the mathematical formula is $\left(1-\beta )(p_{C}-c_{m}-c_{r}+\delta \tau_{Y})(Q-\alpha p_{Y}\right)$. Meanwhile, manufacturers also have to afford part of the cost expense of eco-design, and it is $\frac{\gamma k\tau_{C}^{2}}{2}$. This is the revenue-sharing and cost-sharing contract in this study, where β, γ∈[0, 1]

(15) $$\pi_{CS}=(1-\beta )(p_{C}-c_{m}-c_{s}+\delta \tau_{Y})(Q-\alpha p_{Y})-\frac{(1-\gamma )k\tau_{C}^{2}}{2}$$

(16) $$\pi_{CM}=\beta (p_{C}-c_{m}-c_{s}+\delta \tau_{Y})(Q-\alpha p_{Y})-\frac{\gamma k\tau_{C}^{2}}{2}$$

By solving the above two decision functions (see Equations (15) and (16)), we can get Equation (17):

(17) $$\tau_{C}^{**}=\frac{\gamma \delta (Q-\alpha c_{m}-\alpha c_{s})}{2\gamma k-\alpha \beta \delta^{2}},\;p_{C}^{**}=\frac{\gamma k(Q+\alpha c_{m}+\alpha c_{s})-\alpha \beta \delta^{2}Q}{\alpha (2\gamma k-\alpha \beta \delta^{2})}$$

By comparing and analyzing $\tau_{C}^{*},\;p_{C}^{*}$ under the centralized decision-making condition in Table 1, it shows that β=γ, $\tau_{C}^{**}=\tau_{C}^{*},\;p_{C}^{**}=p_{C}^{*}$. Hence, the supply chain realizes coordination. Furthermore, when the supply chain realizes coordination, it is also necessary to meet the following conditions to ensure the contract’s validity, $\pi_{CS}^{*}\geq \pi_{YS}^{*},\;\pi_{CM}^{*}\geq \pi_{YM}^{*}$. That is:

(18) $$(1-\beta )\frac{k{\left(Q-\alpha c_{m}-\alpha c_{s}\right)}^{2}}{2\alpha (2k-\alpha \delta^{2})}\geq \frac{k{\left(Q-\alpha c_{m}-\alpha c_{s}\right)}^{2}}{2\alpha (4k-\alpha \delta^{2})}\Leftrightarrow \beta \leq \frac{2k}{4k-\alpha \delta^{2}},$$

(19) $$\beta \frac{k{\left(Q-\alpha c_{m}-\alpha c_{s}\right)}^{2}}{2\alpha (2k-\alpha \delta^{2})}\geq \frac{k^{2}{\left(Q-\alpha c_{m}-\alpha c_{s}\right)}^{2}}{\alpha {\left(4k-\alpha \delta^{2}\right)}^{2}}\Leftrightarrow \beta \geq \frac{2k(2k-\alpha \delta^{2})}{{\left(4k-\alpha \delta^{2}\right)}^{2}}.$$

Based on the conclusions above (see Equations (18) and (19)), Conclusion 7 is proved. That is a supply chain coordination contract.

**Conclusion** **7.** By the revenue-sharing contract, the revenue of the supply chain can achieve overall optimality, where β=γ and $\beta \in [\frac{2k(2k-\alpha \delta^{2})}{{\left(4k-\alpha \delta^{2}\right)}^{2}},\;\frac{2k}{4k-\alpha \delta^{2}}]$.

Contract design can coordinate the benefit of the upstream and downstream of the supply chain, which enables suppliers to implement eco-design. In terms of revenue realization, suppliers choose to increase the price of raw materials to transfer part of the eco-design cost to manufacturers. And through revenue-sharing and cost- sharing, risks are reduced and economic benefits are increased. Hence, the manufacturer is more willing to implement eco-design. And it needs to appropriately encourage suppliers to participate in eco-design. The result is to realize the environmental and economic benefits of reducing energy consumption and pollutant emission and provide flexibility for retail price decisions.

Management suggestion 4: When the supplier decides the unit price of component, it should be equal to its unit production cost of component. However, the manufacturer must allocate its profit to the supplier in a certain proportion, and the proportion should be within a certain range. For example, it is like the following case of the production process of rubber tires in numerical experiments.

## 6. Numerical Analysis

To further analyze the effect of eco-design costs on the optimal solution, this article takes the supply chain of tire production as an example. A manufacturer Y produces rubber auxiliaries and additives, which are mainly used in the tire production process for downstream tire company L. In 2019, L Tire Company initiated a green supply-chain management project, and Y Company participated in the project to strengthen the cooperative relationship by responding to the call of customers. During the implementation process for the project, Y Company takes the design of resorcinol (which is included in the list of carcinogens by the World Health Organization. However, China has not completely banned it and it is involved in the chemical reaction of the tire production process, and it thus exists in the tire product) and resorcinol resin substitutes as a breakthrough point. After a series of investments and hard work, the company successfully designed related products by technical scheme screening, small-scale experiment verification, customer small-scale experiment verification, and customer pilot-scale experiment verification, etc. Y Company has invested more than 1 million in the design and development process of substitutes and organized a large number of R&D personnel to tackle key problems. According to the calculation of the same output, the eco-designed products can directly save more than 200,000 in raw material costs each year for customers and generate cost savings, including reducing water consumption, toxic and hazardous substance discharge, and sewage treatment fees. During the design process, L Company supported Y Company and participated in the process (providing test workshops, sharing relevant data, promising to expand procurement and partial cost sharing, etc.). Finally, it made the implementation of eco-design successful.

Based on this case and following studies of Karray et al. [42] and Xia et al. [37], this research sets Q=10,000, α=3, $c_{m}=100,\;c_{s}=50$ (these numerical values do not affect results of the analysis and the graph trend). In the process of numerical analysis, the coefficient of supplier eco-design effort level and the revenue coefficient of manufacturer eco-design show dynamic changes, they have a certain effect on decision-making choice and as a result, we can explore the effects of these two coefficients on eco-design effort level, raw material price, unit retail product price of the manufacturer, product sales volume, and the total revenue under different decision-making conditions. The following discussions are within the boundary conditions of eco-design.

**Figure 3:**
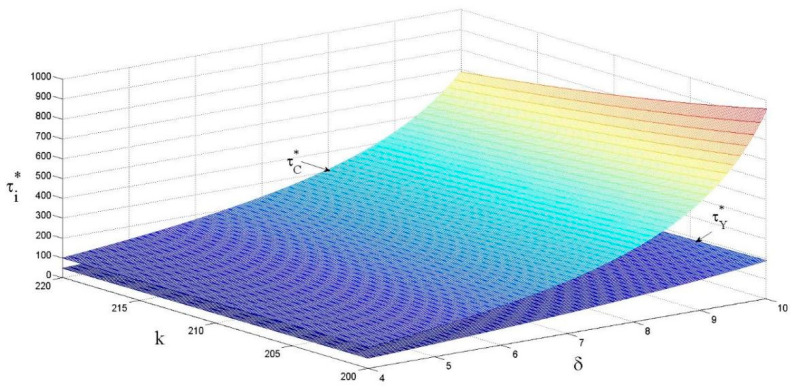
The effects of k and δ on the eco-design effort level.

As shown in Figure 3, the eco-design effort level is the greatest in the centralized decision-making condition. The eco-design effort level is positively correlated with the eco-design revenue coefficient and negatively correlated with the eco-design cost coefficient. The graph trend shows that as the revenue of unit product brought by the eco-design is greater, the trend of eco-design effort level changes in the centralized decision-making condition is more prominent. And the overall trend of changes under the decentralized decision-making condition is more moderate. The effect of eco-design revenue coefficient on the eco-design effort level is relatively small under the decentralized decision-making condition.

**Figure 4:**
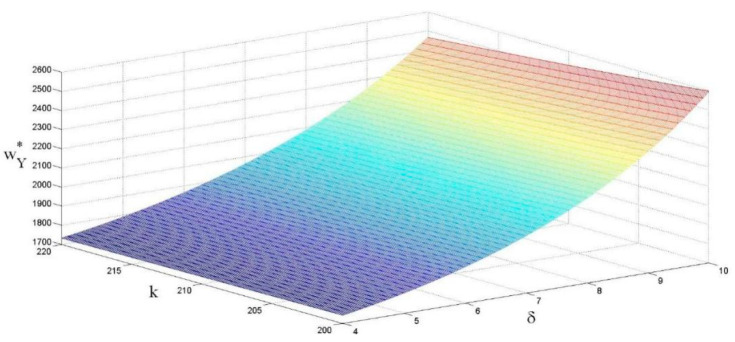
The effects of k and δ on the price of raw materials.

As shown in Figure 4, the unit price of raw materials is positively correlated with the eco-design revenue coefficient, and negatively correlated with the eco-design cost coefficient. The main reason is that when the eco-design revenue coefficient becomes larger, eco-design brings more revenue to the manufacturer. Suppliers will increase the unit price of raw material to make up for the increased cost of the eco-design. Indeed, if the cost coefficient of the eco-design becomes larger, the enthusiasm of suppliers for implementing eco-design will decrease, which indirectly reduces the cost of eco-design and ultimately reduces the unit price of raw materials.

**Figure 5:**
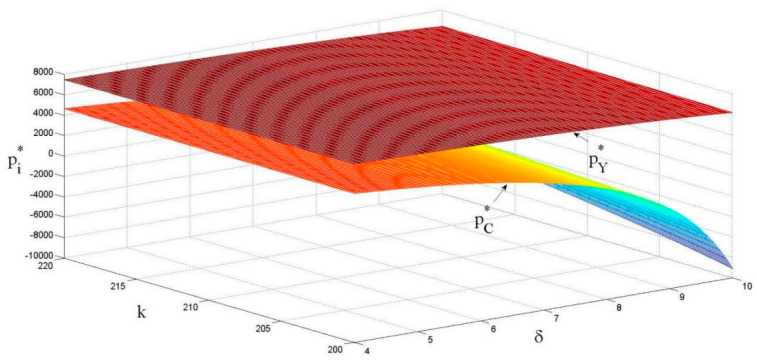
The effects of k and δ on the unit retail price of products.

As shown in Figure 5, the unit retail price of products is positively correlated with the eco-design cost coefficient, and negatively correlated with the eco-design revenue coefficient. The main reason is that when the cost coefficient of eco-design becomes larger, the cost of eco-design increases. For supplier, they will increase the price of raw materials per unit to reduce costs, which directly causes manufacturers to increase the unit retail price of products. When the unit product revenue coefficient of eco-design becomes larger, the eco-design reduces the unit production cost of products (such as energy consumption and material consumption cost), which can increase the manufacturer’s profit margin and create room for price reduction. Therefore, manufacturers will choose to reduce the unit retail price of a product to increase product sales volume to obtain more revenue.

**Figure 6:**
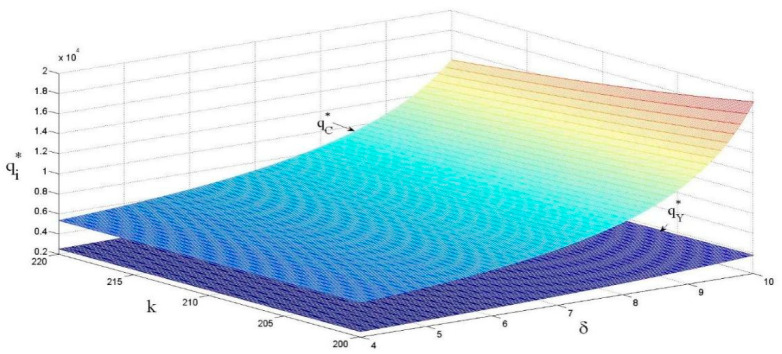
The effects of k and δ on the product sales volume.

Combined with the analysis in Figure 5, Figure 6 shows that product sales volume is positively correlated with the eco-design revenue coefficient and negatively correlated with the eco-design cost coefficient. The main reason for this is that, when the revenue coefficient of eco-design becomes larger, the unit retail price of a product decreases, leading to an increase in product sales volume. On the other hand, when the cost coefficient of eco-design becomes larger, the enthusiasm of a supplier to implement eco-design will decrease, which will reduce the revenue of the manufacturer. The manufacturer will increase the unit retail price of a product to balance the cost, which will finally lead to a decrease in product sales volume.

**Figure 7:**
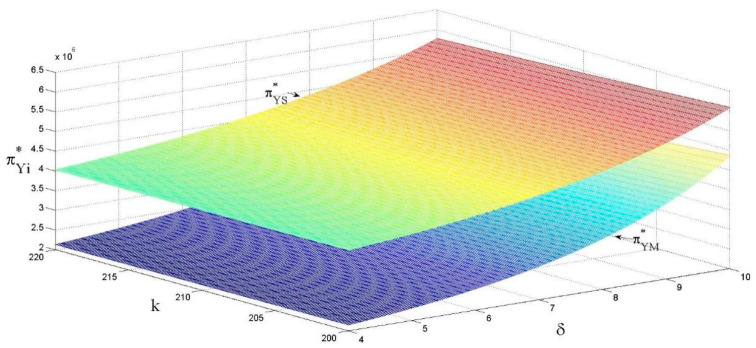
The effects of k and δ on the revenue in decentralized decision-making.

**Figure 8:**
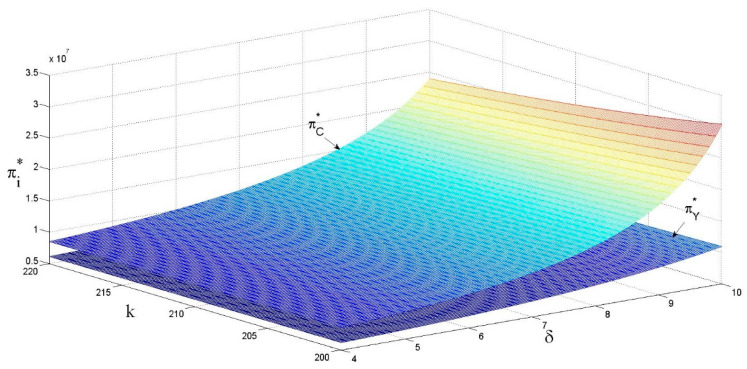
The effects of k and δ on the revenue in centralized decision-making.

As shown in Figure 7 and Figure 8, the revenue of supply chain members is positively correlated with the eco-design revenue coefficient and negatively correlated with the eco-design cost coefficient. No detailed proof will be given here, based on above analysis of the effects on the unit retail price of the product and sales volume.

## 7. Conclusions

This study proposes an innovative eco-design mode in the production processes to achieve energy conservation and emissions reduction. Based on our study, we take three decision-making conditions of supplier’s eco-design in the supply chain into consideration and compare the optimal solutions of variables in different conditions using game theory. Based on it, we realize supply chain coordination by constructing revenue-sharing and cost-sharing contracts, which constructs a multi-win situation among the suppliers, manufacturers, and consumers.

(1) Under eco-design conditions, manufacturers benefit from the decline in the overall cost of products brought by eco-design, which will reduce the selling price of products to improve sales volume and further increase the profit of manufacturers and suppliers. Consumers will also benefit from the price reduction, forming a win-win situation where the three parties benefit together. And if they use decentralized decision-making, the range of suppliers willing to implement eco-design is wider than that in centralized decision-making. However, the effort of eco-design and profit under centralized decision-making is the best.

(2) Manufacturers and suppliers need to formulate reasonable revenue-sharing and cost-sharing contracts for coordination to achieve the optimal overall benefits. And in centralized decision-making condition, the contracts will encourage suppliers to implement eco-design and make the overall economic benefits of the supply chain reach the optimal value.

(3) The cost coefficient of eco-design is positively correlated with the unit price of raw material and the retail price of manufacturer’s products, and negatively correlated with the product sales volume and revenue based on the simulation analysis. The revenue coefficient of manufacture is positively correlated with the unit price of raw material, product vector, and revenue and negatively correlated with the unit retail price of products based on the simulation analysis. 

This study proposes and proves the feasibility and application value of this mode of supplier eco-design to realize the manufacturer’s energy conservation and emissions reduction. It provides practical suggestions for the manufacturer to implement green supply-chain management and green transformation. However, the paper still has some limitations. Firstly, the dynamic impact of eco-design efforts on the manufacturer’s unit product profit lacks a quantitative description, such as some key innovations are realized after the investment efforts, which will increase the profit. Still, it may also form the marginal diminishing effect of the eco-design effort, the reduced profit. The paper only conducts a simulation analysis on the manufacturer’s profit coefficient δ, and preliminarily discusses the impact of its change on the degree of eco-design effort. Further research needs to expand on this. Secondly, it coordinates supply chain cooperation based on revenue-sharing and cost-sharing contracts without considering the free-riding phenomenon of suppliers and multiple customers, which should be considered in follow-up research. In addition, the degree of cooperation between suppliers and manufacturers will be affected by the degree of information-sharing and the relationship between the two sides on the basis of previous cooperation.

## Figures and Tables

**Table 1:** Three eco-design situations and decision sequence and decision variables in the supplier chain.

Condition	Decision Sequence	Decision Variables
1 no eco-design	1supplier	unit price of raw material
	2 manufacturer	unit retail price of products
2 eco-design	1supplier	effort level of eco-design unit price of raw material
	2 manufacturer	unit retail price of products
3 concentrated decision-making		unit price of raw material, effort level of eco-design, unit retail price of products

**Table 2:** The comparison of the optimal solutions of variables in three eco-design situations.

Symbol	Condition N	Condition Y	Condition C
$\tau_{i}^{*}$	―	$\frac{\delta (Q-\alpha c_{m}-\alpha c_{s})}{4k-\alpha \delta^{2}}$	$\frac{\delta (Q-\alpha c_{m}-\alpha c_{s})}{2k-\alpha \delta^{2}}$
$w_{i}^{*}$	$\frac{Q-\alpha c_{m}+\alpha c_{s}}{2\alpha }$	$\frac{2k(Q-\alpha c_{m}+\alpha c_{s})-\alpha^{2}\delta^{2}c_{s}}{\alpha (4k-\alpha \delta^{2})}$	―
$p_{i}^{*}$	$\frac{3Q+\alpha c_{m}+\alpha c_{s}}{4\alpha }$	$\frac{k(3Q+\alpha c_{m}+\alpha c_{s})-\alpha \delta^{2}Q}{\alpha (4k-\alpha \delta^{2})}$	$\frac{k(Q+\alpha c_{m}+\alpha c_{s})-\alpha \delta^{2}Q}{\alpha (2k-\alpha \delta^{2})}$
$q_{i}^{*}$	$\frac{Q-\alpha c_{m}-\alpha c_{s}}{4}$	$\frac{k(Q-\alpha c_{m}-\alpha c_{s})}{4k-\alpha \delta^{2}}$	$\frac{k(Q-\alpha c_{m}-\alpha c_{s})}{2k-\alpha \delta^{2}}$
$\pi_{iS}^{*}$	$\frac{{\left(Q-\alpha c_{m}-\alpha c_{s}\right)}^{2}}{8\alpha }$	$\frac{k{\left(Q-\alpha c_{m}-\alpha c_{s}\right)}^{2}}{2\alpha (4k-\alpha \delta^{2})}$	―
$\pi_{iM}^{*}$	$\frac{{\left(Q-\alpha c_{m}-\alpha c_{s}\right)}^{2}}{16\alpha }$	$\frac{k^{2}{\left(Q-\alpha c_{m}-\alpha c_{s}\right)}^{2}}{\alpha {\left(4k-\alpha \delta^{2}\right)}^{2}}$	―
$\pi_{C}^{*}$	―	―	$\frac{k{\left(Q-\alpha c_{m}-\alpha c_{s}\right)}^{2}}{2\alpha (2k-\alpha \delta^{2})}$

## Data Availability

The data was taken from a Chinese manufacturer, Safe-Run Company.
